# Influence of *Helicobacter pylori* infection on risk of rheumatoid arthritis: a nationwide population-based study

**DOI:** 10.1038/s41598-023-42207-w

**Published:** 2023-09-13

**Authors:** Tzu-Hsuan Lee, Meng-Che Wu, Ming-Hung Lee, Pei-Lun Liao, Chieh-Chung Lin, James Cheng-Chung Wei

**Affiliations:** 1 Division of Gastroenterology, Children’s Medical Center, Taichung Veterans General Hospital, Taichung, Taiwan; 2 School of Medicine, Chung Shan Medical University, Taichung, Taiwan; 3 Department of Post-Baccalaureate, Medicine College of Medicine, National Chung Hsing University, Taichung, Taiwan; 4 Pediatric Inflammatory Bowel Disease Center, Massachusetts General Hospital, Boston, MA USA; 5 Department of Otolaryngology-Head & Neck Surgery, Taichung Veterans General Hospital, Taichung, Taiwan; 6 Department of Medical Research, Chung Shan Medical University Hospital, Taichung, Taiwan; 7 Institute of Medicine, Chung Shan Medical University, No. 110, Sec 1, Jianguo N. Road, Taichung, 40201 Taiwan; 8 Department of Nursing, Chung Shan Medical University, Taichung, Taiwan; 9 Department of Allergy, Immunology and Rheumatology, Chung Shan Medical University Hospital, Taichung, Taiwan; 10 Graduate Institute of Integrated Medicine, China Medical University, Taichung, Taiwan

**Keywords:** Rheumatoid arthritis, Bacterial host response

## Abstract

The relationship between *Helicobacter pylori* infection and rheumatoid arthritis has been investigated, but the results remain controversial. This study aims to determine the association between the two diseases via a 17-year retrospective cohort study. Using the National Health Insurance Research Database, a nationwide population based in Taiwan, we identified 97,533 individuals with *H. pylori* infection and matched controls between 2000 and 2017 using propensity score matching at a 1:1 ratio. The adjusted hazard ratio of rheumatoid arthritis was determined by multiple Cox regression. The incidence rate of rheumatoid arthritis was 1.28 per 10,000 person-months in the *H. pylori* cohort, with a higher risk compared to the control group. In the < 30 years old subgroup, the risk was highest, especially in women < 30 years old with *H. pylori* infection. Patients with < 1 year follow-up showed 1.58 times higher susceptibility to rheumatoid arthritis. Individuals with follow-ups of 1–5 years and over 5 years demonstrated 1.43 and 1.44 times higher risks of rheumatoid arthritis, respectively. Our study showed *H. pylori* infection was associated with the development of rheumatoid arthritis. Clinicians should note higher risk, especially < 30 years old. More research needed to understand underlying mechanism.

## Introduction

Rheumatoid arthritis (RA) is a chronic autoimmune systemic inflammatory disorder that involves synovial joints primarily. The main clinical features of RA include the articular manifestations of swelling, pain, and stiffness in joints^1,2^. Failure to treat patients and late-stage disease states can destroy joints due to uncontrolled inflammation damage to cartilage and bone, eventually leading to joint deformities and disability; furthermore, various systemic and extraarticular complications could be significant and prominent^3–5^. However, the definitive cause of RA remains unknown. RA is theorized to develop when a genetically susceptible individual experiences an environmental stimulus such as exposure to infections that trigger an autoimmune reaction^6–8^.

Helicobacter *pylori* (*H. pylori*) is a gram-negative, spiral-shaped, flagellated, microaerophilic, extracellular bacterium, and is one of the most common chronic bacterial infections in humans^9,10^. Immune activation occurs after persistent *H. pylori* infection and is manifested by continuous cytokine signaling from epithelial cells and infiltration of gastric mucosa by neutrophils, macrophages, lymphocytes, and the generation of antibodies and effector T cells with both Th1 and Th2 components^11^. Chronic *H. pylori* infection was found to be a source of persistent antigenic stimulation and experiments have shown that *H. pylori* infection is related to several autoimmune diseases involving alterations of gastric autoimmunity. These findings imply that RA may also have a similar pathogenesis^12–16^.

Although a few studies have been published reviewing the relationship between *H. pylori* infection and RA, some found a positive correlation, but others showed the opposite results^13,14,17^. Given the conflicting results regarding RA risk among *H. pylori* infection patients, and due to the challenge of various disabilities and systemic complications resulting from RA, as well as the need for early identification of RA, we were interested in determining the relationship between RA and *H. pylori* and any related factors. In this study, we investigated the relationship between *H. pylori* infection and RA by analyzing a nationwide, longitudinal population-based retrospective cohort in Taiwan.

## Methods

### Data source

**Figure 1:**
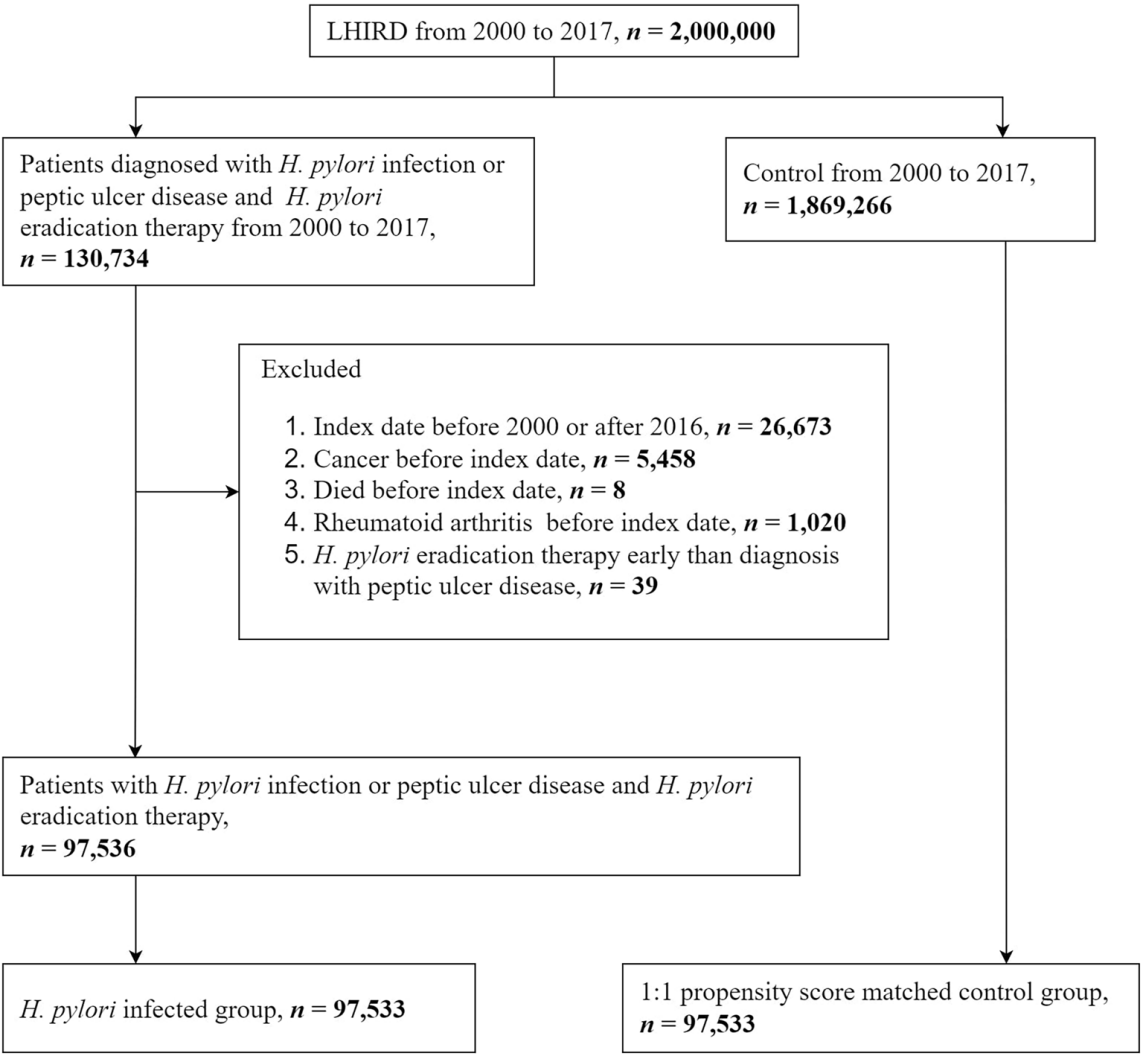
Study flow chart.

The retrospective cohort study aims to investigate the relationship between *H. pylori* infection and RA. The flow diagram is displayed in Fig. 1. The Longitudinal Health Insurance Research Database (LHID) contains 2,000,000 individuals that are sampled randomly from the National Health Insurance Research Database (NHIRD), which is a national population-based insurance system enrolling over ninety-nine percent population in Taiwan and comprises medical data that date back to 1997^18,19^. In addition, the LHID is a large and reliable database that has been extensively used in numerous studies published worldwide. Patients' diagnoses are documented following the International Classification of diseases, both the 9th (ICD-9-CM) and 10th Revision, Clinical Modification (ICD-10-CM). The LHID also involves demographic data, claims for hospital and hospital expenditures, and other patients’ clinical information. Propensity score matching was done for chosen variables to control the deviations to avoid confounding biases.

A large number of studies have been published using this database. All methodologies were executed in strict adherence to pertinent guidelines and regulations. This research was conducted in alignment with the principles outlined in the Declaration of Helsinki. The personal data of all participants underwent thorough de-identification throughout the study. Furthermore, the study was approved by the Chung Shan Medical University Hospital Institution Review Committee (IRB number CS1-20201) and informed consent is waved by the committee since retrospective nature of study.

### Study population

We identified individuals who received a diagnosis of *H. pylori* infection spanning the years 2000 to 2017, employing the following criteria: *H. pylori* infection (ICD-9-CM: 041.86, ICD-10-CM: B96.81), gastrointestinal tract hemorrhage (ICD-9-CM: 578.9, ICD-10-CM: K92.0-92.2), or peptic ulcer (ICD-9-CM: 531–534; ICD-10-CM: K25-28), and had subsequently undergone *H. pylori* eradication therapy. Verification of *H. pylori* treatment was achieved through upper endoscopic and biopsy-based assessment, in adherence to the National Health Insurance system guidelines. This verification process encompassed histological evaluation, rapid urease testing, or biopsy culture. The administration of anti-*H. pylori* treatment encompassed both the standard triple therapy (consisting of H2 receptor antagonists or proton pump inhibitors, tetracycline or amoxicillin, and clarithromycin or metronidazole) and the bismuth-containing quadruple therapy. The application of the drug combination followed a consistent protocol lasting 1–2 weeks for *H. pylori* eradication^10^.

The index date, for the purpose of this study, was established as the earliest date of *H. pylori* infection diagnosis, gastrointestinal tract hemorrhage diagnosis, or peptic ulcer diagnosis. Exclusions from the study comprised patients with pre-existing medical histories of RA, cancer, or documented deaths prior to the index date, along with cases diagnosed before the year 2000, individuals who had previously received *H. pylori* eradication therapy prior to the index date, and instances where a suitable control, matched by propensity score, could not be identified.

The control cohort was comprised of individuals registered within the LHID who had not undergone anti-*H. pylori* treatment. To counterbalance the potential influence of confounding bias arising from comorbidities on the incidence of RA, control participants were subjected to a 1:1 propensity score-based matching process with *H. pylori*-infected patients, employing an 8-to-1 digit greedy matching algorithm. For the control group, the index date was harmonized with the corresponding matched cases. In this intricate process, a logistic regression model was deployed to predict the probability, incorporating variables such as age, sex, urbanization level, insurance type, and comorbidities within the year preceding the index date. Following the propensity score matching, the standardized differences in covariates between the two groups were revealed to be below 0.1, thus affirming a satisfactory balance between the cohorts. Ultimately, the analysis encompassed a total of 97,533 patients who underwent *H. pylori* eradication therapy, matched one-to-one with control individuals based on propensity scores, resulting in an equivalent number of participants in each group, namely 97,533.

### Outcome and comorbidities

We identified RA patients by searching the ICD-9-CM code 714.0, and ICD-10-CM codes M05 and M06, and the RA diagnostic accuracy was determined by incorporating patients with a previous diagnosis code, which included at least one hospitalization or three outpatient visits. Comorbidities related to RA were listed. We selected the following comorbidities: hypertension, diabetes mellitus, hyperlipidemia, asthma, cerebrovascular disease, chronic pulmonary diseases, chronic kidney disease, chronic liver disease, pneumonia, osteoporosis, Sjogren’s syndrome, systemic lupus erythematosus (SLE), and ankylosing spondylitis. All comorbidities were defined by having at least hospitalization for one time or outpatient visits two times for a particular ICD code in 2 years prior to the index date. The comorbidities were applied as covariates in the multivariate analysis.

### Statistical analysis

Descriptive statistics were harnessed to scrutinize the distribution of foundational characteristics. In order to mitigate the potential bias arising from measured factors, a meticulous 1:1 propensity score matching (PSM) was meticulously carried out, employing a method of greedy nearest-neighbor, non-replacement matching, with a precisely defined caliper width of 0.01. The assessment of disparities in covariates between the two cohorts was facilitated by the application of the absolute standardized difference (ASD). A value of ASD lower than 0.10 serves as an indicator of equilibrium in the characteristics of the studied groups^20^.

The incidence rate of study events, along with their corresponding 95% confidence intervals (CIs), was computed utilizing the normal approximation to the Poisson distribution. The cumulative probability of RA was evaluated through the utilization of Kaplan–Meier analysis^21^. The discernment of variations in Kaplan–Meier curves between the study groups was accomplished via a log-rank test. Employing Cox proportional hazard analysis, the estimation of the hazard ratio (HR) for RA was undertaken. This endeavor encompassed multifaceted Cox proportional hazard regression models, encompassing parameters such as age, sex, urbanization level, insurance type, and comorbidities.

Sensitivity analysis was conducted to explore the association between *H. pylori* infection and RA risk, stratified by follow-up time (within 0–12 months, 12–60 months, and beyond 60 months from the index date), as well as by sex and age. All statistical analyses were executed utilizing SAS software version 9.4. A significance level denoted by *p* < 0.05 was held as the threshold for statistical significance.

### Ethics approval and consent to participate

The study was approved by the Chung Shan Medical University Hospital Institution Review Committee (IRB number CS1-20201). Since the large database contains over 2 million individuals, and all data derived from the LHID were de-identification, the consent to participants is not applicable.

## Results

After employing propensity score matching, the distribution of selected parameters including index year, age, sex, urbanization, social status, insurance coverage, and comorbidities showed close balance between the two cohorts (Table 1). The average follow-up duration was approximately 126 months for individuals in the *H. pylori* group and 122 months for those in the control group. To assess the monthly incidence rates of RA, diagnoses were categorized by the month they were identified. The results demonstrated a significant difference in the incidence rate of RA between the two groups, with the *H. pylori* group experiencing a higher rate compared to the control group (1.28 vs. 0.89 per 10,000 person-months respectively, adjusted hazard ratio (aHR): 1.45, 95% confidence interval (CI) 1.34–1.56, Table 2).

**Table 1:** Baseline characteristics of study groups after propensity score matching.

	After propensity score matching		
	Control (*n* = 97,533)	*H. pylori*-infected group (*n* = 97,533)	ASD^a^
Index year			
2001–2005	49,950 (51.21%)	50,057 (51.32%)	< 0.0001
2006–2010	27,224 (27.91%)	27,184 (27.87%)	
2011–2016	20,359 (20.87%)	20,292 (20.81%)	
Sex			
Female	42,283 (43.35%)	42,301 (43.37%)	0.0004
Male	55,250 (56.65%)	55,232 (56.63%)	
Age			
< 30	10,289 (10.55%)	10,819 (11.09%)	0.0302
30–44	28,044 (28.75%)	28,115 (28.83%)	
45–64	44,523 (45.65%)	44,195 (45.31%)	
≥ 65	14,677 (15.05%)	14,404 (14.77%)	
Mean ± SD	48.63 ± 14.77	48.39 ± 14.83	
Urbanization			
Urban	60,480 (62.01%)	60,374 (61.9%)	< 0.0001
Sub-urban	28,996 (29.73%)	29,025 (29.76%)	
Rural	8057 (8.26%)	8134 (8.34%)	
Insurance property			
Government employee insurance	7341 (7.53%)	7344 (7.53%)	0.0827
Labor insurance	60,137 (61.66%)	59,956 (61.47%)	
Farmer/fisherman/water resources employee insurance	14,591 (14.96%)	14,581 (14.95%)	
Low-income family	344 (0.35%)	376 (0.39%)	
Local insurance by government	13,650 (14%)	13,749 (14.1%)	
Others	1470 (1.51%)	1527 (1.57%)	
Comorbidity			
Hypertension	20,979 (21.51%)	20,899 (21.43%)	0.0020
Diabetes mellitus	10,209 (10.47%)	10,188 (10.45%)	0.0007
Hyperlipidemia	13,311 (13.65%)	13,275 (13.61%)	0.0011
Asthma	3698 (3.79%)	3702 (3.8%)	0.0002
Cerebrovascular disease	3668 (3.76%)	3767 (3.86%)	0.0053
Chronic pulmonary diseases	9822 (10.07%)	9868 (10.12%)	0.0016
Chronic kidney disease	2855 (2.93%)	2883 (2.96%)	0.0017
Chronic liver disease	15,748 (16.15%)	15,690 (16.09%)	0.0016
Pneumonia	2068 (2.12%)	2128 (2.18%)	0.0042
Osteoporosis	2889 (2.96%)	2895 (2.97%)	0.0004
Sjogren’s syndrome	370 (0.38%)	421 (0.43%)	0.0082
Systemic lupus erythematosus	90 (0.09%)	99 (0.1%)	0.0030
Ankylosing spondylitis	359 (0.37%)	399 (0.41%)	0.0066

^a^*ASD,* absolute standard difference.

**Table 2:** Incidence of RA in the study group.

	After propensity score matching	
	Control (*n* = 97,533)	*H. pylori*-infected group (*n* = 97,533)
Follow-up person months	11,961,822	12,290,765
New case	1061	1574
Incidence rate^a^ (95% CI)	0.89 (0.84–0.94)	1.28 (1.22–1.35)
Crude relative risk (95% CI)	Reference	1.44 (1.33–1.56)
aHR^b^ (95% CI)	Reference	1.45 (1.34–1.56)

^a^Incidence rate, per 10,000 person months.

^b^*aHR,* adjusted hazard ratio, the demographic variables (such as sex, age, urbanization, insurance property), and comorbidities were included in the multiple Cox regression.

Our findings, derived from the propensity score matched data, indicated that the risk of RA was notably elevated in the *H. pylori* infection group, extending to specific subgroups such as individuals with low socioeconomic status, women, the elderly, and patients with comorbidities like chronic kidney disease, chronic liver disease, osteoporosis, and other autoimmune disorders such as Sjogren’s syndrome, SLE, and ankylosing spondylitis (Table 3). Even after applying multivariate adjustment, the group with *H. pylori* infection still exhibited an increased risk of RA (aHR: 1.45, 95% CI 1.34–1.56, as shown in Table 3).

**Table 3:** Cox proportional hazard model analysis for risk of RA.

	After propensity score matching	
	aHR^a^ (95% CI)	*p*-value
*H. pylori*-infected group (*ref*: control)	1.45 (1.34–1.56)	< 0.0001
Index year		
2001–2005	Reference	
2006–2010	0.74 (0.67–0.81)	< 0.0001
2011–2016	0.63 (0.54–0.74)	< 0.0001
Sex		
Female	Reference	
Male	0.44 (0.41–0.48)	< 0.0001
Age		
< 30	Reference	
30–44	2.24 (1.81–2.79)	< 0.0001
45–64	3.77 (3.06–4.64)	< 0.0001
≥ 65	4.2 (3.35–5.28)	< 0.0001
Urbanization		
Urban	0.9 (0.82–0.99)	0.0246
Sub-urban	Reference	
Rural	1.06 (0.91–1.24)	0.4325
Insurance property		
Government employee insurance	1.03 (0.89–1.18)	0.7329
Labor insurance	Reference	
Farmer/fisherman/water resources employee insurance	0.91 (0.8–1.03)	0.1363
Low-income family	1.26 (0.67–2.34)	0.4744
Local insurance by government	1.08 (0.96–1.21)	0.1969
Others	1.1 (0.82–1.48)	0.5406
Comorbidity (*ref*: without comorbidity)		
Hypertension	1.05 (0.96–1.16)	0.2960
Diabetes mellitus	0.89 (0.78–1.01)	0.0756
Hyperlipidemia	1.12 (1–1.25)	0.0528
Asthma	1 (0.81–1.23)	0.9986
Cerebrovascular disease	0.94 (0.77–1.14)	0.5331
Chronic pulmonary diseases	1.24 (1.08–1.42)	0.0019
Kidney disease	1.38 (1.14–1.67)	0.0009
Liver disease	1.3 (1.18–1.43)	< 0.0001
Pneumonia	1.38 (1.1–1.73)	0.0057
Osteoporosis	1.38 (1.19–1.6)	< 0.0001
Sjogren syndrome	2.15 (1.5–3.1)	< 0.0001
Systemic lupus erythematosus	3.31 (1.95–5.64)	< 0.0001
Ankylosing spondylitis	2.74 (1.86–4.04)	< 0.0001

^a^*aHR,* adjusted hazard ratio.

Further elaboration from sensitivity analysis indicated that patients below the age of 30, with H. pylori infection, exhibited a notably higher inclination towards RA (aHR: 2.19, 95% CI 1.41–3.38). Sequentially, the risk tendencies were observed in the 45–64 years age group (1.5 times higher risk), followed by the 30–44 years age group (1.4 times higher risk), and finally the ≥ 65 years age group (1.25 times higher risk). Additionally, it was noted that the highest RA risk occurred within the first year following H. pylori diagnosis (aHR: 1.58, 95% CI 1.26–1.99), with a subsequent risk of 1.43 within 1–5 years and 1.44 after 5 years, all of which displayed statistical significance (Table 4).

**Table 4:** Sensitivity analysis for the aHRs stratified by follow-up time interval, sex, and age groups.

	N	Incidence rate (95% CI) of RA		aHR^a^ (95% CI)
		Control	*H. pylori*-infected group	
Follow-up time interval				
Index date to 12 months	195,066	1.03 (0.86–1.23)	1.62 (1.41–1.87)	1.58 (1.26–1.99)
12–60 months	192,757	0.76 (0.69–0.84)	1.08 (1–1.18)	1.43 (1.25–1.62)
> 60 months	151,851	0.97 (0.89–1.06)	1.39 (1.3–1.49)	1.44 (1.29–1.61)
*p* for interaction				0.9919
Sex				
Female	84,584	1.29 (1.2–1.39)	1.9 (1.79–2.02)	1.48 (1.34–1.63)
Male	110,482	0.56 (0.51–0.62)	0.78 (0.72–0.85)	1.38 (1.21–1.58)
*p* for interaction				0.4744
Age on the index date				
< 30	21,108	0.2 (0.14–0.29)	0.45 (0.35–0.57)	2.19 (1.41–3.38)
30–44	56,159	0.62 (0.55–0.71)	0.88 (0.79–0.98)	1.40 (1.18–1.65)
45–64	88,718	1.09 (1–1.18)	1.63 (1.52–1.74)	1.50 (1.35–1.67)
≥ 65	29,081	1.48 (1.3–1.69)	1.83 (1.63–2.06)	1.25 (1.05–1.49)
*p* for interaction				0.0860

^a^*aHR,* adjusted hazard ratio.

**Figure 2:**
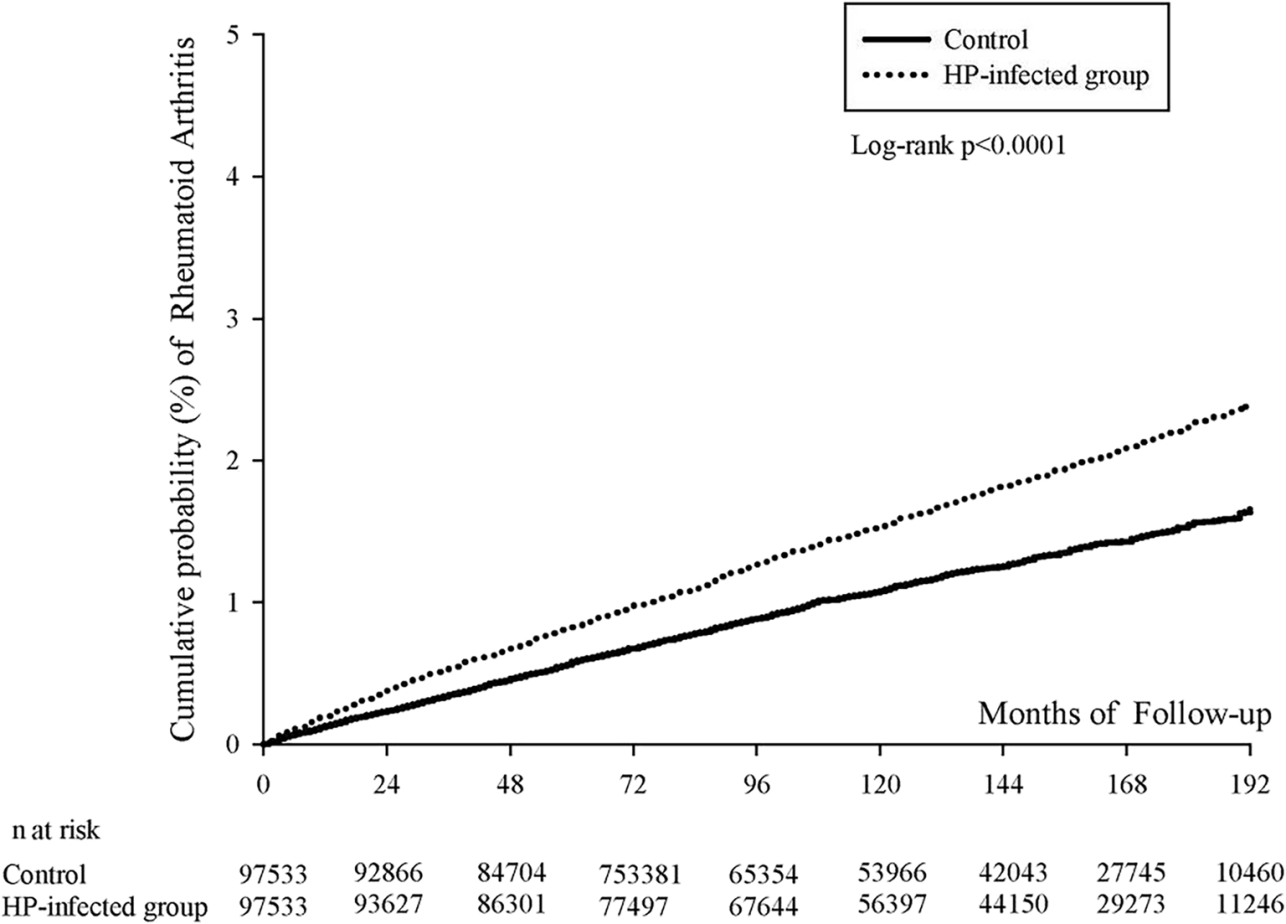
Kaplan–Meier curves of cumulative probability of RA in study groups. HP, *Helicobacter pylori.*

Upon delving into age-sex subgroup analysis (Table 5), the highest adjusted hazard ratio was evident among female patients under 30 years of age (2.22, 95% CI 1.26–3.91), followed by male patients of the same age category (2.12, 95% CI 1.07–4.20). To visually represent the cumulative probability of RA, Fig. 2 displayed the Kaplan–Meier curves.

**Table 5:** RA risk in patients with *H. pylori* infection stratified by age-sex subgroups.

Age	Sex	N	Incidence rate (95% CI) of RA		aHR^a^ (95% CI)
			Control	*H. pylori*-infected group	
< 30	Female	8924	0.28 (0.18–0.46)	0.65 (0.48–0.88)	2.22 (1.26–3.91)
< 30	Male	12,184	0.15 (0.08–0.26)	0.30 (0.21–0.44)	2.12 (1.07–4.20)
30–44	Female	22,962	1.01 (0.86–1.18)	1.50 (1.32–1.71)	1.47 (1.20–1.80)
30–44	Male	33,197	0.35 (0.28–0.43)	0.43 (0.35–0.53)	1.23 (0.91–1.67)
45–64	Female	39,756	1.55 (1.4–1.71)	2.37 (2.18–2.56)	1.52 (1.34–1.73)
45–64	Male	48,962	0.68 (0.59–0.78)	1.00 (0.89–1.12)	1.46 (1.22–1.76)
≥ 65	Female	12,942	1.84 (1.55–2.19)	2.29 (1.96–2.66)	1.25 (0.99–1.57)
≥ 65	Male	16,139	1.16 (0.94–1.42)	1.45 (1.21–1.73)	1.25 (0.95–1.64)

^a^*aHR,* adjusted hazard ratio.

## Discussion

According to our study findings, the development of RA was statistically associated with *H. pylori* infection. The incidence rate of RA was significantly higher in the *H. pylori*-infected group than in the non-infected group during the follow-up interval, which ranged from the first year to more than 5 years. Intriguingly, the incidence rate of RA increased more significantly in patients with *H. pylori* infection under the age of 30, in both males and females. To date, this is the first and the largest cohort of a national, longitudinal, population-based dataset to reveal a higher risk of RA among *H. pylori*-infected patients. The epidemiological interpretation of this association might provide insight into the pathogenesis of RA. In addition, physicians should remain vigilant about the risk of RA in patients with *H. pylori* infection.

Genetic and environmental influences such as infections are believed to interact and trigger adaptive responses associated with autoimmunity and have been implicated in the development of RA^22^. *H. pylori* has been intensely studied over the past three decades to determine the characteristics involved in instigating host immunity that results in some autoimmune diseases. Various mechanisms have been proposed, including chronic inflammatory progress, polyclonal lymphocyte activation, molecular mimicry, superantigens, epitope spreading, bystander activation, endothelial cell damage, and highly immune-dominant virulence^23,24^.

The virulent factors of *H. pylori* in the development of autoimmune diseases have attracted widespread attention over the last two decades. According to the literature, *H. pylori* possesses several virulence factors. Two crucial markers of virulence have been widely researched in *H. pylori*, including vacuolating cytotoxin A (VacA), which leads to epithelial cell damage, and cytotoxin-associated gene A (CagA), which is translocated into the gastric epithelium, endowing it with an increased intensity of gastric inflammation by increasing the secretion of pro-inflammatory cytokines^25–28^. Several scholars have suggested that the relationships between the infectious agent and the autoimmunity response may differ depending on the virulence of the infecting strain. Infection with *H. pylori* CagA-positive strains was associated with more serious inflammatory reactions and an increase in the risk of adverse clinical consequences. *H. pylori* virulence factors had antigenic similarity with host proteins antigens and were involved in the development of autoimmune diseases through alternative intracellular signaling pathways, which modulated and dysregulated the host immune response^13^.

Hamed et al. investigated the effects of *H. pylori*’s existence and activity on RA disease activity. According to their results, patients with active RA (DAS-28 > 3.2) showed a higher positive rate of *H. pylori* stool antigen than patients in remission^29^. Ebrahimia et al. showed that RA patients who were *H. pylori* CagA-positive tended to have severe clinical manifestations with higher DAS-28 scores and higher mean VAS scores than CagA-negative patients. Furthermore, in their study, the laboratory indices of activity including rheumatoid factor (RF), erythrocyte sedimentation rate (ESR), C-reactive protein (CRP), anti-cyclic citrullinated peptide (Anti.CCP), and anti-mutated citrullinated vimentin (Anti-MCV) were significantly higher in patients with *H. pylori*-positive than in the negative patients^30^. A report by Zentilin et al. found similar findings that *H. pylori*-eradicated RA patients demonstrated obvious improvement over time in clinical parameters such as joint symptoms, functional ability, and progressive decreases in laboratory indices, such as ESR, CRP, and antinuclear antibody compared with *H. pylori*-negative RA patients^31^. That is, *H. pylori* infection might be one of the pathogenesis of RA. Whereas other studies demonstrated different trends. Nakamura et al. have shown that RA patients with *H. pylori* infection have lower values of rheumatoid factor compared with *H. pylori*-negative in RA patients^32^. Ishikawa et al. showed *H. pylori* infection was not associated with increased disease activity in patients with RA treated with NSAIDs^33^. Further prospective research with more participants is required to reach a definitive conclusion.

The correlation and mechanisms of *H. pylori* infection in the pathogenesis of RA are still controversial. Yamanishi et al. found that purified urease by *H. pylori* chronically stimulates splenic B cells in vitro, causing the production of antigen-specific conventional antibodies such as IgM-type rheumatoid factor^34^. Moreover, some research has reported that *H. pylori* induces antigen-stimulated T-cell activation and interferes with the maturation of dendritic cells, resulting in a tolerogenic phenotype. The initial steps of the autoimmune response in RA are likely to involve environmental triggers on mucosal surfaces, such as exposure to cigarette smoke in the airway, oral bacteria, specifically *Porphyromonas gingivalis*, resulting in periodontitis^35,36^, and even the gut microbiota^37^. Furthermore, a variety of investigations have attempted to elucidate the association between the gut microbiome and the development of RA^38^. Studies of stool samples and culture-independent microbiota have demonstrated significant enrichment of the *Prevotellaceae* bacterial family, especially *Prevotella *spp. in patients in pre-clinical stages of RA compared with controls, which demonstrates that the gastrointestinal microbiome may act as an environmental modulator that could dysregulate the autoimmune response, eventually leading to RA^39^.

The interaction of *H. pylori* with the gastrointestinal microbiome and its role in dysbiosis leading to the development of autoimmune arthritis have recently attracted much attention. It has been shown that *H. pylori* commonly colonizes and changes the microbiota in the upper gastrointestinal tract^31,40–42^. *H. pylori* may cause large intestinal microbiota changes through stomach immunopathogenesis, including hypochlorhydria and hypergastrinemia^43^. Moreover, a study on the microbial 16S ribosomal RNA gene of gastric biopsies and stool samples by Gu et al. found that *H. pylori* infection is a pivotal factor in gastric and gut dysbiosis, which changes the microbial diversity and community structure, and is possibly involved in gastric carcinogenesis^44^. Block et al. reported the mechanisms whereby the gut microbiota regulates arthritis development by modulating follicular helper T (Tfh) cell differentiation^45^. In addition, in an animal study using SKG mice harboring a microbiota dominated by *Prevotella copri* from RA patients, interleukin-17 (IL-17) was increased with a higher number of intestinal Th17 cells, which response to the arthritis-related autoantigen 60S ribosomal protein L23a (RPL23A) in the lymphocytes of regional lymph nodes and the colon, leading to the development of arthritis. According to their results, dysbiosis increases arthritis sensitivity by activating autoreactive T cells in the intestine^46^. Both T follicular helper cells and IL-17 are produced by Th17 or mast cells appearing in the RA synovium and are crucial mediators of inflammation in RA pathogenesis^47–51^.

Thus, the gastrointestinal microbiota is hypothesized to play a contributory role in the pathogenesis of RA via the gut-joint axis. Concerning the mechanisms underlying our study results, we speculate that the increased incidence of RA in *H. pylori*-infected patients may be caused by a chronic inflammatory response, immune dysregulation, and autoimmune reaction through *H. pylori* infection, and may also even involve dysbiosis of the gastrointestinal microbiome, which could serve as a trigger of environmental factors in the development of RA. Further studies of the precise mechanism by which *H. pylori* affect the gastrointestinal microbiome and the dysregulation of immunological response in the development of RA are warranted.

In previous epidemiological and clinical studies, data on the prevalence of *H. pylori* infection between patients with RA and the general population are inconclusive. Ebrahimia et al. found that 57.9% of RA patients had *H. pylori* antigens^30^. A cohort study by Tanaka et al., which enrolled 1815 RA patients, showed that there was a lower prevalence of *H. pylori* infection with a total of 871 (49.3%) positive *H. pylori* serum antibodies in RA patients compared with healthy Japanese individuals^52^. For each group of age, the rate of *H. pylori* seropositivity increased with age, ranging from 24.5% in RA patients under 30 years old to 56.5% in patients over 60 years. These results are consistent with recent research on the epidemiology of *H. pylori*, which reported that the prevalence of *H. pylori* infection in the general population increases with age^53,54^. Nevertheless, the prevalence of *H. pylori* infection varies among children and adults and in different regions and continents worldwide^55^. Early exposure to *H. pylori* in childhood has been observed because intrafamilial transmission is common, especially in children under the age of 15^56^. *H. pylori* infection probably develops in children and chronic infection continues into adulthood until it is tested and the bacterium is eradicated^10^. In our study, patients under 30 years of age at the index date had a remarkably higher risk of RA. Therefore, we should be aware of possible related joint symptoms in patients diagnosed with *H. pylori* at a younger age, because early diagnosis is key to optimal therapeutic success.

Studies on the relationship between *H. pylori* and RA have yielded contradictory findings in recent decades. In contrast to our results, Smyk et al. noted that the evidence supporting *H. pylori* infection as a cause of RA is less convincing^14^. A systematic review and meta-analysis study by Youssefi et al. showed no significant correlation between *H. pylori* infection and RA^13^. A similar finding was also reported in a cross-sectional and historical cohort study by L. E. Bartels et al. who evaluated a large population-based database of individuals in Denmark with 56,000 patients diagnosed as *H. pylori*-positive or -negative by urea breath test to explore a possible causative link between *H. pylori* and the development of RA, with a median follow-up of 8.2 years. Their results reported 390 patients were diagnosed with RA via the urea breath test and there was no significant association between the prevalence of RA and *H. pylori* infection, with an RA prevalence of 0.66% among *H. pylori*-positive patients compared to an RA prevalence of 0.71% among *H. pylori*-negative patients. During follow-up after the urea breath test, 299 patients developed RA. The study also demonstrated a comparable incidence of new RA cases between *H. pylori*-positive and -negative individuals (0.54% vs. 0.69%), which fit in with an adjusted HR of 0.80 (95% CI 0.56–1.13). That is, these findings did not support any connection between *H. pylori* infection and RA^17^. Our findings were the opposite of those reported in the population-based study in Denmark, which may be due to differences in immunological responses to *H. pylori* antigen among different ethnic populations, genetic differences, including human leukocyte antigen, the prevalence of *H. pylori* in different areas, and *H. pylori* strain heterogeneity in different geographic regions. Moreover, the criteria for diagnosis, follow-up interval, and sensitivity of the registry system in different studies may not have been the same. In line with our study, Etchegaray-Morales suggested there may have been links between *H. pylori* infection, with *H. pylori* playing a pivotal role as an environmental trigger, and the development of RA^14^.

Our study has several methodological strengths. First, the study cohort was obtained from the NHIRD, and so the sample size was large, with approximately 195,000 patients enrolled in this study, and the data were nationally representative, with a minimal likelihood of loss to follow-up or recall and selection bias. Second, we used propensity score matching to control and minimize potential confounders.

However, our study has several limitations. First, detailed information about family history, marriage, body weight index, physical activities, inflammatory biomarkers, such as rheumatoid factor or anti-citrullinated peptide antibody, and personal habits, such as dietary habits, cigarette use, and alcohol use are not collected in the NHIRD. It has been assumed that the composition of the gut microbiota, which is closely linked to lifestyle habits, may act as an environmental trigger in the development of RA. Our results might have been biased by these unmeasured confounding factors. In light of this, we focused on factors present in the database, including index year, age, sex, urbanization, social status, insurance properties, and comorbidities. These factors were used for propensity score matching to establish balanced cohorts for our analysis. Nevertheless, these lacking parameters could serve as avenues for more in-depth future research. Secondly, using the NHIRD database, we analyzed patients with *H. pylori*-related diagnoses and patients receiving anti-*H. pylori* treatment, but the severity and duration of *H. pylori* infection could not be measured in this study. The NHIRD does not contain data on *H. pylori* virulence factors. Therefore, the Cag A-positive *H. pylori* strain could not be identified, which could have revealed a potential association with an increased risk of developing RA and adverse clinical outcomes. The awareness of symptoms of *H. pylori* infection and RA among the public and general practitioners varies considerably, which could have affected patients presenting for care as well as the diagnosis by clinicians. Hence, the prevalence of *H. pylori* infection could have been underestimated, which would have influenced the incidence rate of RA. Furthermore, it was not possible to establish whether anti-*H. pylori* therapy could reduce RA risks, and it could not be determined whether *H. pylori* eradication may be capable of inducing improvement in RA disease activity in infected patients. Moreover, due to limitations within the NHIRD, we were unable to images, obtain histopathological reports, or laboratory serum test results for certain conditions such as autoimmune atrophy gastritis (requiring histopathological finding, macrocytic anemia and serum cobalamin level) and autoimmune pancreatitis (requiring image, histopathological finding, and IgG4 confirmation). As a result, these specific diagnoses were not included in our analysis. Hence, our research focused on major systemic autoimmune diseases. Other potentially related autoimmune diseases are areas of future exploration. Finally, the question of whether our study’s results are generally applicable to other ethnic groups remains unclear, as most patients were Taiwanese. Future research is needed to gain a deeper understanding of the possible mechanisms of these associations.

## Conclusions

*H. pylori* infections were associated with the development of rheumatoid arthritis. Clinicians should be aware of the increased risk of developing rheumatoid arthritis among patients with *H. pylori* infection, particularly in patients under 30 years of age. Further investigation is needed to clarify the basic mechanism of the association.

## Data Availability

The datasets generated and analyzed during the current study are not publicly available due to privacy purposes but are available from the corresponding author upon reasonable request.

## Abbreviations

*H. pylori*: Helicobacter pylori
RA: Rheumatoid arthritis
LHID: Longitudinal health insurance research database
NHIRD: National health insurance research database
ICD: International classification of diseases
